# Insomnia and creativity in Chinese adolescents: mediation through need for cognition

**DOI:** 10.1186/s40359-024-01663-3

**Published:** 2024-03-29

**Authors:** Xiaoyang Ren, Min Shi, Si Si

**Affiliations:** Department of Psychology, Shandong Normal University, No.88 East Wenhua Road, 250014 Jinan, China

**Keywords:** Creativity, Insomnia, Need for cognition, Adolescent, Time-of-day effect, Indirect effect

## Abstract

**Background:**

Creativity is an essential cognitive ability that plays a crucial role in advanced thinking. While previous research has demonstrated the impact of insomnia on cognitive function, its effects on creativity in Chinese adolescents remain unclear. This study explored the relationship between insomnia (specifically, daytime and nighttime disturbances) and creativity in adolescents. Additionally, it examined the potential mediating effect of the need for cognition on this relationship.

**Methods:**

Questionnaires were administered to 302 adolescents to measure their creativity, need for cognition, and insomnia levels using the Williams Creative Tendencies Scale, Need for Cognition Scale, and Bergen Insomnia Scale, respectively. Regression analysis was conducted to examine the direct impact of insomnia on creativity. Furthermore, a mediation model was constructed to investigate the role of the need for cognition in mediating the relationship between insomnia and creativity.

**Results:**

The findings of the present study indicated that insomnia had a direct impact on the creativity of adolescents, demonstrating a time-of-day effect. Daytime disturbances were found to have a positive correlation with overall creativity and imagination, whereas no significant direct effect was found between nighttime disturbances and creativity. Further analysis revealed that insomnia, specifically daytime disturbances, might influence creativity by affecting the individual’s need for cognition. However, no similar indirect effects were observed for the relationship between nighttime disturbances and creativity.

**Conclusions:**

Our findings indicate that adolescents might experience improved creativity as a result of daytime disruptions, and the level of need for cognition could play a crucial role in understanding the link between insomnia and creativity in adolescents.

**Supplementary Information:**

The online version contains supplementary material available at 10.1186/s40359-024-01663-3.

## Introduction

Insomnia is a condition characterized by an individual’s self-reported difficulties in sleeping [1, 2]. It is characterized by symptoms such as taking a long time to fall asleep, waking up frequently during the night, experiencing prolonged periods of wakefulness during sleep, and frequent brief awakenings [3]. In recent years, issues like staying up late, not getting enough sleep, and struggling to fall asleep have become increasingly prevalent among adolescents [4]. The White Paper 2023 China Youth and Children’s Sleep Index, released by the China Sleep Research Association, reveals concerning statistics about the sleep patterns of junior high school students in China. According to the report, only 18.9% of these students manage to sleep for more than 8 h, while a staggering 59.4% sleep for less than 7 h. On average, these students only get 6.82 h of sleep, indicating that the majority of them do not meet the recommended amount of sleep. A study conducted in the Shandong Province of China explored the prevalence of sleep problems among adolescents. The findings revealed that 37.44% of adolescents suffered from insufficient sleep, while 26.89% reported experiencing poor sleep quality [5]. Another meta-analysis, which included 63 studies and a total of 430,422 Chinese adolescents, discovered that 104,802 adolescents experienced sleep disturbances. The overall prevalence of sleep problems was found to be 26%, with junior high school students having a detection rate of 20% [6]. As widely known, adolescents go through a crucial stage of psychological transformation. Issues such as sleep deprivation and sleep disorders appear to have a significant influence on their mental well-being, especially in terms of cognition and personality development [7, 8].

Can tired minds generate creative ideas? Some researchers have found that the cognitive processes utilized before sleep by individuals with insomnia, such as rehearsing, planning, and problem-solving, are similar to the stages involved in creative thinking, such as preparation and incubation [9, 10]. As a result, a hypothesis has emerged suggesting that individuals with disrupted sleep might exhibit greater creativity. In addition, it should be noted that disrupted sleep and the widely recognized consequences of sleep deprivation are symptoms of depression and anxiety [11, 12], while depression and anxiety have also been associated with creativity [13]. This suggests that sleep issues could have been prevalent among individuals who are highly creative. However, it is important to consider that sleep problems have been shown to negatively affect cognitive function as well. For example, a study using fMRI have demonstrated that lack of sleep reduced the communication between various brain regions such as the amygdala, dorsolateral prefrontal cortex, dorsal anterior cingulate gyrus, and right inferior frontal gyrus. This weakened functional connectivity could result in a negative bias when it comes to encoding memories [14]. Additionally, research has found that sleep deprivation could also impact the activity of brain regions involved in fearful learning, namely the prefrontal cortex, hippocampus, and amygdala [15]. Since the activity of the aforementioned brain regions is crucial for individual creativity, some researchers have also suggested that problems such as sleep deprivation and sleep disorders may produce impairments in cognition, memory, etc., which in turn interfere with creativity [16].

It is noteworthy that only two studies have delved into the connection between insomnia and creativity until now. Firstly, researchers discovered a positive correlation between insomnia and creativity by comparing the prevalence of sleep disturbances in 30 creative children versus 30 control children. Notably, the highly creative children exhibited a higher incidence of sleep disturbances than the control group [17]. Subsequently, a recent study indicated a minor direct impact of a global insomnia factor on divergent thinking, implying time-of-day effects where nighttime sleep disturbances positively predicted divergent thinking more strongly than daytime disturbances [18]. These findings suggest that sleep disturbances may possess some beneficial predictive effects on creativity among children and adolescents. However, there could be disparities in the impact of sleep disturbances during the day and night. Despite this, the majority of existing studies have focused on the influence of insomnia on creative thinking, leaving a gap in research evidence regarding its effects on creative personality. It is established that insomnia is linked to personality traits [19]. Therefore, the primary objective of this study is to investigate the relationship between adolescents’ creativity (specifically creative personality) and insomnia. Building on the outcomes of previous studies, we hypothesized that insomnia would significantly and positively predict adolescents’ creativity (creative personality).

Although a tenuous link has been established between insomnia and creativity, it is postulated that additional variables might influence this relationship. Taking these observations into account, a crucial question arises: How does insomnia impact creativity? Since coming up with original and useful ideas requires several cognitively demanding processes [20, 21], the need for cognition may also play an important role in creativity. The need for cognition refers to an individual’s tendency to engage in and derive pleasure from tasks that require cognitive effort [22]. Individuals with a strong need for cognition are more prone to innovate and have a deeper interest in addressing challenging problems. For instance, research suggested that those with a high cognitive need were more likely to generate ideas for ambiguous scenarios [23]. Furthermore, individuals with a strong need for cognition exhibited heightened creativity in problem-solving and possessed more pronounced creative personalities [24, 25]. Therefore, the need for cognition might serve as a significant and positive predictor of creative personality [26]. In considering the role of insomnia in creativity, it is plausible that the need for cognition could act as a mediator, influencing the association between the two variables.

However, there was evidence that insomnia could impact individuals’ willingness to invest more time and effort when faced with complex tasks. The microanalytic model of insomnia highlighted hyperarousal as a key regulatory feature, which could distort perceptions of time and exacerbate the challenges associated with falling asleep and experiencing distress. As a result, the consequences of insomnia on the following day could include fatigue, mood disturbances such as irritability, cognitive impairments, and a reduced ability to engage in or enjoy mentally demanding tasks [27]. Furthermore, the maintained cognitive model of insomnia suggested that insomniacs tend to worry excessively about sleep and its consequences. This negative cognition leaded to emotional distress, and the resulting anxiety prompted individuals to hyperfocus on internal and external cues related to sleep-related threats. Consequently, this state of anxiety could lead to a lack of interest and motivation in solving complex problems, as well as crowding out the time needed for engaging in mentally challenging tasks [28]. Supported by neuroimaging and neurobiochemistry evidence, researchers have found that individuals with insomnia often exhibit impairments in various cognitive functions, including episodic memory, working memory, and certain aspects of executive functioning [7]. Given these findings, it is likely that insomnia can reduce an individual’s cognitive engagement and motivation to seek new knowledge, thereby suppressing the anticipated effect of insomnia on creativity. Therefore, the second objective of our study was to further investigate the psychological mechanisms that underlie the impact of insomnia on creativity. Drawing from the aforementioned theoretical and empirical evidence, we hypothesized that the need for cognition played a mediating role in the relationship between insomnia and creativity.

Taking into account that previous research primarily focused on young adults or children, who exhibited distinct sleep patterns compared to adolescents, the relationship between insomnia and the creativity of adolescents, particularly their creative personality, remained enigmatic. The objective of this study was to explore the impact of insomnia on adolescents’ creativity, specifically their creative personality, and to unravel the underlying mechanisms. Drawing from existing theoretical and empirical research, we postulated that: (1) insomnia, encompassing both daytime and nighttime disturbances, was associated with creativity in adolescents, and there might exist time-of-day effects (H1); and (2) the need for cognition might serve as a mediator between insomnia and creativity (H2).

## Materials and methods

### Design

After a thorough literature review and consideration of previous research, the research questions and hypotheses were formulated in January 2023. Utilizing a cross-sectional research design, questionnaires were administered to a cohort of middle school students in Jinan, Shandong Province, in April of the same year. These questionnaires aimed to capture data on all the relevant research variables, including creativity, insomnia, and the need for cognition at the same time. Subsequently, the collected data was entered into a database and subjected to rigorous checking and analysis.

### Participants and procedure

To ensure the validity and relevance of our study, we collaborated closely with a local school in the recruitment process. Initially, we liaised with the school’s head to disseminate recruitment details. Leveraging the assistance of class teachers, we carefully selected participants based on the following criteria: all participants were required to be native Chinese speakers with normal or corrected vision, exhibit no signs of mental or physical health issues, possess normal intellectual development, not encounter any reading difficulties, and not consume psychotropic drugs. Only students who expressed a willingness to participate and fulfilled the study’s criteria were ultimately chosen to participate in the testing process. This meticulous approach ensured that our sample population was representative and well-suited for the objectives of our research.

In this study, 318 junior high school students participated, of whom 302 were included in the primary analysis due to having complete datasets, yielding an effective participation rate of 94.97%. Participants’ ages ranged from 12 to 14, with an average of 12.97 years (*SD* = 0.49). Specifically, 41 were 12 years old, 229 were 13, and 32 were 14. 147 were females (48.7%) and 155 were males (51.3%). Regarding the parents’ educational backgrounds, the survey revealed that 31 fathers (10.3%) and 34 mothers (11.3%) held university degrees or higher qualifications. Notably, most parents had completed their education at the middle or high school level (70.2%). When it came to parental occupations, the survey found that the fathers’ top three professions were doctors (25.5%), self-employed individuals (11.6%), and drivers (8.3%). Meanwhile, for mothers, the most common occupations were self-employed (19.2%), salespeople (11.6%), and laborers (8.9%).

The Institutional Review Board of Shandong Normal University has granted approval for this study, ensuring that all measurements adhere strictly to the pertinent guidelines and regulations for psychological research. The group tests were conducted within the classroom setting, led by a psychology-major researcher as the primary tester. Initially, we secured the authorization and support of the school’s teaching department. Subsequently, we utilized the students’ self-study period to clarify the purpose of the research and underscore the principles of voluntariness, anonymity, and honesty. Ultimately, the participants were required to complete a psychological test within approximately 30 min, assessing various aspects such as creativity, need for cognition, insomnia, along with personal family information.

### Measures

#### Creativity

An adapted version of the Williams Creative Tendencies Scale (WCTS) was utilized to assess the creativity of the participants [29]. This scale was widely employed in numerous prior creativity studies and exhibited strong reliability [30, 31]. The adapted version included 11 items to measure adventurousness, 14 items to measure curiosity, 13 items to measure imagination, and 12 items to measure challenge. Each item was rated on a Likert scale ranging from 1 (strongly disagree) to 3 (strongly agree). By compiling the total scores, we can effectively evaluate the creativity of the students. Notably, all the items demonstrated good reliability, with a Cronbach’s α value of 0.86.

#### Need for cognition

The Need for Cognition Scale (NCS) [22] was employed in its shortened version to assess participants’ need for cognition. The 18-item Chinese version of the NCS was initially introduced [32] and subsequently validated as suitable for both adolescents and young adults in subsequent studies [33]. Participants were instructed to answer the questions based on their actual circumstances. Each item was rated using a Likert-type scale ranging from 1 (strongly opposed) to 5 (strongly agreed). The total score was calculated by summing up the responses to all 18 items, with higher scores indicating a stronger need for cognition. This measurement demonstrated good reliability in the current study, with a Cronbach’s α value of 0.76.

#### Insomnia

We utilized the Bergen Insomnia Scale (BIS) to assess insomnia among the participants [34]. The scale comprises six items, all aligned with the Diagnostic and Statistical Manual of Mental Disorders (DSM-IV-TR) criteria for clinical insomnia. The validity of these items has been confirmed through subjective reports and polysomnographic data, encompassing sleep-stage progression, limb movement, and physiological measurements of respiration during controlled laboratory sleep. Three items focus on nighttime disturbances, such as “How many days a week did it take you over 30 minutes to fall asleep after switching off the lights in the past month?”; the other three items target daytime disturbances, like “How many days a week did you feel rested upon waking up in the past month?”. Participants were asked to rate their symptoms on a weekly basis using an eight-point scale ranging from 0 to 7. The total score for the first three items represents nighttime disturbances, while the last three items reflect daytime disturbances. This measurement demonstrated strong reliability for daytime disturbances (Cronbach’s α = 0.86), nighttime disturbances (Cronbach’s α = 0.62), and the overall insomnia score (Cronbach’s α = 0.80) in the Chinese population. According to previous literature, this scale demonstrated good reliability among the Chinese population [35].

#### Socioeconomic status (SES)

Recognizing the challenges in precisely measuring income, domestic researchers often turn to an alternative method: assessing a family’s socioeconomic status (SES) through a detailed analysis of their parents’ occupation and education level [36]. In the present study, we utilized the SES questionnaire to gather participants’ reports on their parents’ occupational and educational backgrounds [37]. These reports were then coded and graded, following established occupational classification standards, to ensure consistency and comparability across respondents. The occupational classification system employed in this study encompassed five distinct levels: (1) those engaged in temporary, unskilled, agricultural, or non-technical work; (2) self-employed individuals, manual laborers, and technicians; (3) general management and professional technical personnel, including clerks and employees in the commercial service industry; (4) middle-level professionals, managers, and technical personnel, as well as auxiliary professionals specializing in various fields of science, technology, and enterprise work; and (5) senior professional technicians, executives, and leading cadres exercising administrative functions in government, institutions, and social organizations, as well as high- and middle-level managers in large and medium-sized enterprises and private business owners. By utilizing this graded classification system, we aimed to capture a comprehensive representation of participants’ SES backgrounds, ensuring the validity and reliability of our findings.

Furthermore, the educational attainment of parents was categorized into distinct levels:“no schooling or primary education”, “junior middle school”, “high school or technical secondary school”, “junior college”, “university (undergraduate)”, and “graduate”. Participants were required to select the most appropriate category based on their parents’ educational qualifications, and each choice was assigned a numerical score ranging from 1 to 6 during the coding process.

Ultimately, the cumulative score served as an indicator of the family’s socioeconomic status, with a potential range spanning from 4 to 22. Notably, in this research endeavor, the SES scores for both mothers and fathers were computed separately, allowing for a nuanced understanding of each parent’s contribution to the overall socioeconomic profile of the family.

### Data analysis

First, we employed the Pearson correlation to assess the relationships between the research variables in the present study. To explore the direct impact of insomnia (independent variables) on creativity (dependent variables), we resorted to multiple linear regression analysis. Specifically, gender, age, socioeconomic status of both parents, and insomnia total score (or daytime and nighttime disturbances) were simultaneously entered into the regression equation. Additionally, we utilized the mediation model to delve into the intricate relationships between insomnia, need for cognition, and creativity. To validate the mediation effects, we relied on the bootstrapping method. From the data, 5000 bootstrap samples were drawn, and 95% bootstrap confidence intervals (CI) were computed. For these statistical analyses, we employed SPSS 17.0 process SPSS macro PROCESS (model 4) (http://www.afhayes.com) [38]. This macro has been extensively used and developed for testing complex models incorporating mediating variables [39].

## Results

### Common method deviation test

While the self-report method is a popular choice for data collection, it can potentially lead to common method variance (CMV) issues. To mitigate these concerns, we implemented various control measures to safeguard participants’ anonymity. Among these measures, we ensured that the collected data was strictly limited to scientific research purposes and employed reverse expressions for certain items [40]. Additionally, to enhance the study’s precision, we utilized the Harman single factor test to process the data. Specifically, we conducted a non-rotating principal component factor analysis on the aforementioned items. The results indicated that the first factor explained only 13.66% of the variation (falling below the 40% threshold). Consequently, this study did not exhibit significant common method variance issues in the collected data.

### Descriptive statistics of study variables

Table 1 presents the means, standard deviations, bivariate correlations and gender differences among study variables. The independent samples t-test results revealed that females significantly scored higher than males on measures of insomnia, daytime disturbances, and imagination. Our findings further indicated a positive correlation between insomnia and daytime disturbances with imagination, whereas a negative correlation was observed with the need for cognition. Moreover, the need for cognition demonstrated positive associations with the creativity total score, adventure, curiosity, imagination, and challenge. Mother’s socioeconomic status (SES) exhibited a positive association with imagination. The data for all variables had no outliers and were within three standard deviations. The distributions of all variables approached normality, with skewness and kurtosis ranging from − 1 to 1.

### Direct effect tests

The collinearity diagnosis revealed that the tolerance values for the variables of insomnia, daytime and nighttime disturbances, and need for cognition were greater than 0.2, ranging from 0.78 to 0.98, indicating the absence of significant collinearity issues.

The regression analysis results demonstrated that insomnia (*β* = 0.19, *p* < 0.01) and daytime disturbances (*β* = 0.24, *p* < 0.01) positively predicted imagination when controlling for gender, age, father’s SES, and mother’s SES. However, no significant direct effect of nighttime disturbances was observed on the creativity total score, adversity, curiosity, imagination, and challenge (Tables 2 and 3). Therefore, H1 was supported. Based on previous research, effect sizes of 0.10, 0.30, and 0.50 are considered small, medium, and large, respectively [18, 41]. Consequently, insomnia (*β* = 0.19) and daytime disturbances (*β* = 0.24) exhibited small-to-medium positive effects on creativity, particularly in terms of imagination.

**Table 1:** Descriptive statistics of study variables

	Bivariate correlations												Total		Females		Males			
Variables	1	2	3	4	5	6	7	8	9	10	11	12	*M*	*SD*	*M*	*SD*	*M*	*SD*	*t*	*p*
1. Age	1	−0.02	−0.01	0.02	0.07	−0.04	0.01	−0.03	−0.04	0.01	−0.06	0.02	12.97	0.49	12.99	0.44	12.95	0.54	0.56	0.576
2. SES_f_		1	0.38^**^	−0.06	−0.08	−0.02	0.07	0.02	0.00	0.03	−0.02	0.06	10.96	5.82	10.95	5.97	10.97	5.69	−0.04	0.966
3. SES_m_			1	0.00	0.05	−0.07	0.06	0.11	0.01	0.08	0.15^*^	0.11	12.58	5.44	13.05	5.60	12.14	5.26	1.47	0.143
4. Insomnia				1	0.89^**^	0.82^**^	−0.13^*^	0.05	−0.06	−0.02	0.23^**^	−0.05	15.64	10.13	18.03	9.60	13.37	10.12	4.10^***^	0.000
5. DD					1	0.47^**^	−0.12^*^	0.10	−0.03	0.02	0.28^**^	0.00	9.53	6.63	11.54	6.15	7.62	6.52	5.38^***^	0.000
6. ND						1	−0.10	−0.03	−0.07	−0.06	0.09	−0.09	6.11	5.17	6.48	5.31	5.75	5.03	1.23	0.218
7. NC							1	0.43^**^	0.37^**^	0.39^**^	0.19^**^	0.49^**^	56.62	9.96	56.61	9.65	56.63	10.27	−0.02	0.986
8. Creativity								1	0.79^**^	0.87^**^	0.80^**^	0.79^**^	103.40	12.82	104.46	12.90	102.40	12.70	1.40	0.164
9. Adventure									1	0.64^**^	0.48^**^	0.57^**^	23.08	3.19	23.19	3.21	22.98	3.18	0.57	0.568
10. Curiosity										1	0.56^**^	0.60^**^	29.85	4.49	29.76	4.49	29.95	4.51	−0.37	0.709
11. Imagination											1	0.49^**^	24.17	4.65	25.23	4.67	23.17	4.42	3.95^***^	0.000
12. Challenge												1	26.29	3.34	26.28	3.38	26.30	3.31	−0.63	0.950

Note^*^p < 0.05, ^**^p < 0.01, ^***^p < 0.001; SES_f_=father socioeconomic status, SES_m_=mother socioeconomic status; Insomnia = DD + ND; DD = daytime disturbances; ND = nighttime disturbances; NC = need for cognition

**Table 2:** Direct effect of insomnia on creativity

Outcome	Predictor	Regression coefficient				Model fit	
		*β*	*95%CI*	*p*		*R*	*R* ^*2*^
Creativity	Gender	−0.06	[−0.36, 0.11]		0.283	0.14	0.02
	Age	−0.03	[−0.14, 0.09]		0.628		
	SES_f_	−0.02	[−0.03, 0.02]		0.720		
	SES_m_	0.11	[−0.00, 0.04]		0.069		
	Insomnia	0.03	[−0.08, 0.15]		0.577		
Adventure	Gender	−0.05	[−0.34, 0.14]		0.403	0.09	0.01
	Age	−0.04	[−0.16, 0.07]		0.479		
	SES_f_	−0.01	[−0.02, 0.02]		0.932		
	SES_m_	0.00	[−0.02, 0.02]		0.976		
	Insomnia	−0.07	[−0.19, 0.05]		0.245		
Curiosity	Gender	0.03	[−0.18, 0.29]		0.670	0.09	0.01
	Age	0.01	[−0.11, 0.12]		0.901		
	SES_f_	−0.00	[−0.02, 0.02]		0.962		
	SES_m_	0.09	[−0.01, 0.04]		0.170		
	Insomnia	−0.02	[−0.13, 0.10]		0.792		
Imagination	Gender	−0.17	[−0.56,−0.11]		0.003	0.33	0.11
	Age	−0.07	[−0.18, 0.04]		0.194		
	SES_f_	−0.07	[−0.03, 0.01]		0.243		
	SES_m_	0.16	[0.01, 0.05]		0.009		
	**Insomnia**	**0.19**	**[0.08, 0.30]**		**0.001**		
Challenge	Gender	0.00	[−0.23, 0.24]		0.974	0.12	0.02
	Age	0.02	[−0.09, 0.14]		0.707		
	SES_f_	0.02	[−0.02, 0.03]		0.747		
	SES_m_	0.10	[−0.00, 0.04]		0.108		
	Insomnia	−0.05	[−0.16, 0.07]		0.428		

*Note* 0 = female, 1 = male; SESf = father socioeconomic status, SESm = mother socioeconomic status

**Table 3:** Direct effect of daytime and nighttime disturbances on creativity

Outcome	Predictor	Regression coefficient			Model fit	
		*β*	*95%CI*	*p*	*R*	*R* ^*2*^
Creativity	Gender	−0.04	[−0.32, 0.15]	0.478	0.17	0.03
	Age	−0.04	[−0.15, 0.08]	0.505		
	SES_f_	−0.01	[−0.02, 0.02]	0.864		
	SES_m_	0.10	[−0.00, 0.04]	0.114		
	DD	0.12	[−0.01, 0.26]	0.076		
	ND	−0.09	[−0.22, 0.04]	0.188		
Adventure	Gender	−0.04	[−0.32, 0.16]	0.493	0.10	0.01
	Age	−0.05	[−0.16, 0.07]	0.439		
	SES_f_	−0.00	[−0.02, 0.02]	0.990		
	SES_m_	−0.00	[−0.02, 0.02]	0.951		
	DD	−0.01	[−0.14, 0.13]	0.934		
	ND	−0.08	[−0.21, 0.05]	0.252		
Curiosity	Gender	0.04	[−0.16, 0.32]	0.501	0.12	0.01
	Age	−0.00	[−0.12, 0.11]	0.992		
	SES_f_	0.01	[−0.02, 0.02]	0.929		
	SES_m_	0.08	[−0.01, 0.04]	0.234		
	DD	0.07	[−0.07, 0.20]	0.352		
	ND	−0.09	[−0.22, 0.05]	0.201		
Imagination	Gender	−0.14	[−0.51, 0.06]	0.013	0.35	0.12
	Age	−0.08	[−0.19,−0.02]	0.126		
	SES_f_	−0.06	[−0.03, 0.01]	0.349		
	SES_m_	−0.14	[0.00, 0.05]	0.020		
	**DD**	**0.24**	**[0.12, 0.37]**	**0.000**		
	ND	−0.03	[−0.15, 0.10]	0.656		
Challenge	Gender	0.02	[−0.20, 0.28]	0.758	0.15	0.02
	Age	0.01	[−0.10, 0.13]	0.819		
	SES_f_	0.03	[−0.02, 0.03]	0.639		
	SES_m_	0.09	[−0.01, 0.04]	0.158		
	DD	0.05	[−0.09, 0.19]	0.471		
	ND	−0.11	[−0.24, 0.02]	0.106		

*Note* 0 = female, 1 = male; SES_f_=father socioeconomic status, SES_m_=mother socioeconomic status; DD = daytime disturbances; ND = nighttime disturbances

### Indirect effect tests

#### The indirect effect of need for cognition between insomnia and creativity

**Fig. 1:**
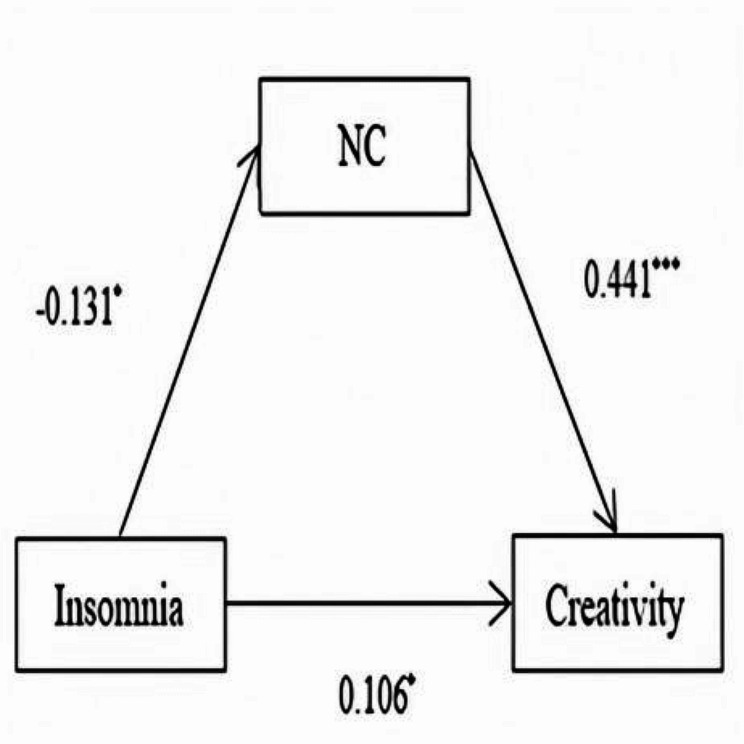
Mediation analysis model testing relationships among insomnia, need for cognition (NC) and creativity

Firstly, the total effect of insomnia on creativity was tested, and it was demonstrated that the path coefficient was not significant. Subsequently, the mediating variable of cognition was added to the model to obtain the path type shown in Fig. 1. The results showed that insomnia had a direct effect on creativity, and the need for cognition played an indirect role between insomnia and creativity (Table 4). The bootstrap test was utilized, and 5000 repeated samples were taken to test the mediating effect and estimate the confidence interval. The absence of 0 in the 95% confidence interval suggested that the indirect effect was significant (see Table 5). Therefore, H2 was supported. Similar results were found for imagination, while only indirect effects were found for adventure, curiosity, and challenge. According to the recently proposed mediation effect test method [42], the indirect effect of need for cognition on the relationship between insomnia and creativity was established, which manifested suppression effects. In other words, the inclusion of the need for cognition enhanced the relationship between insomnia and creativity.

**Table 4:** Indirect effect of need for cognition between insomnia on creativity

Outcome	Predictor	Model fit			Regression coefficient	
		*R*	*R* ^*2*^	*F*	*β*	*t*
NC	Insomnia	0.131	0.017	5.249	-0.131	-2.291^*^
Creativity	Insomnia	0.440	0.194	35.865	**0.106**	2.021^*^
	NC				0.441	8.419^***^
Creativity	Insomnia	0.048	0.002	0.693	**0.048**	0.833
NC	Insomnia	0.131	0.017	5.250	-0.131	-2.291^*^
Adventure	Insomnia	0.373	0.139	24.127	-0.010	-0.181
	NC				0.371	6.860^***^
Adventure	Insomnia	0.059	0.003	1.031	-0.059	-1.015
NC	Insomnia	0.131	0.017	5.250	-0.131	-2.291^*^
Curiosity	Insomnia	0.394	0.155	27.465	0.030	0.567
	NC				0.397	7.400^***^
Curiosity	Insomnia	0.022	0.001	0.140	-0.022	-0.375
NC	Insomnia	0.147	0.022	2.179	-0.137	-2.322^*^
Imagination	Insomnia	0.378	0.143	12.404	**0.219**	3.936^***^
	NC				0.211	3.881^***^
Imagination	Insomnia	0.316	0.100	10.999	**0.191**	3.371^***^
NC	Insomnia	0.131	0.017	5.250	-0.131	-2.291^*^
Challenge	Insomnia	0.492	0.242	47.813	0.016	0.315
	NC				0.494	9.731^***^
Challenge	Insomnia	0.049	0.002	0.717	-0.049	-0.847

*Note* ^*^p < 0.05, ^**^p < 0.01; NC = need for cognition

**Table 5:** Mediating effect of need for cognition between insomnia on creativity

		Effect	BootSE	BootLLCI	BootULCI
Creativity	Direct	0.106	0.052	0.003	0.209
	Indirect	-0.058	0.028	-0.116	-0.005
Adventure	Direct	-0.010	0.054	-0.116	0.097
	Indirect	-0.049	0.024	-0.101	-0.006
Curiosity	Direct	0.030	0.054	-0.075	0.136
	Indirect	-0.052	0.026	-0.109	-0.007
Imagination	Direct	0.219	0.056	0.079	0.302
	Indirect	-0.029	0.015	-0.065	-0.005
Challenge	Direct	0.016	0.051	-0.084	0.116
	Indirect	-0.065	0.031	-0.129	-0.006

*Note* SE = standard error; LLCI = lower limit of confidence interval; ULCI = upper limit of confidence interval

#### The indirect effect of need for cognition between daytime disturbances and creativity

**Fig. 2:**
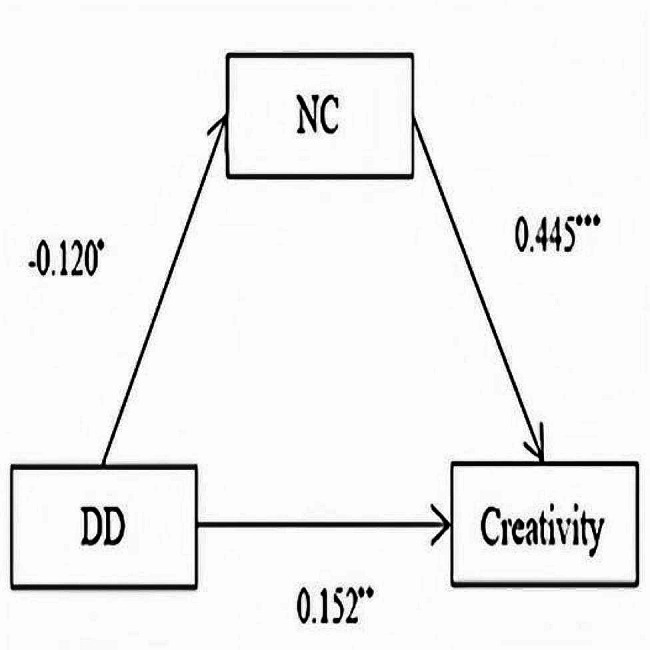
Mediation analysis model testing relationships among daytime disturbances (DD), need for cognition (NC) and creativity

Similar analysis processes were also conducted to investigate the relationship between daytime disturbances and creativity. Testing the total effect of daytime disturbances on creativity revealed that the path coefficient was not significant. The mediating variable, need for cognition, was then added to the model to obtain the path type shown in Fig. 2. The results showed that daytime disturbances had a direct effect on creativity, and the need for cognition played an indirect role in the relationship between daytime disturbances and creativity (Table 6). Finally, the bootstrap test was employed, and 5000 replicated samples were taken to test the mediating effect and establish the confidence interval. The exclusion of 0 from the 95% confidence interval indicated a statistically significant indirect effect (see Table 7). Similar results were found for imagination, while for adventure, curiosity, and challenge, only indirect effects were found. Also, the inclusion of the need for cognition enhanced the relationship between daytime disturbances and creativity.

#### The indirect effect of need for cognition between nighttime disturbances and creativity

Although **s**imilar analysis processes were also conducted to examine the relationship between nighttime disturbances and creativity, no significant direct of nighttime disturbances or indirect effects of the need for cognition were found (see Supplementary Table S1, Table S2).

**Table 6:** Indirect effect of need for cognition between daytime disturbance on creativity

		Model fit			Regression coefficient	
Outcome	Predictor	*R*	*R* ^*2*^	*F*	*β*	*t*
NC	DD	0.120	0.014	4.398	-0.120	-2.097^*^
Creativity	DD	0.453	0.205	38.582	**0.152**	2.920^**^
	NC				0.445	8.576^***^
Creativity	DD	0.098	0.010	2.915	**0.098**	1.707
NC	DD	0.120	0.014	4.398	-0.120	-2.097^*^
Adventure	DD	0.373	0.139	24.142	0.013	0.241
	NC				0.374	6.923^***^
Adventure	DD	0.032	0.001	0.306	-0.032	-0.554
NC	DD	0.120	0.014	4.398	-0.120	-2.097^*^
Curiosity	DD	0.398	0.158	28.132	0.064	1.204
	NC				0.401	7.495^***^
Curiosity	DD	0.016	0.000	0.079	0.016	0.281
NC	DD	0.141	0.020	2.018	-0.133	-2.216^*^
Imagination	DD	0.398	0.158	13.969	**0.258**	4.597^***^
	NC				0.214	3.975^***^
Imagination	DD	0.337	0.114	12.728	**0.230**	4.026^***^
NC	DD	0.120	0.014	4.398	-0.120	-2.097^*^
Challenge	DD	0.495	0.245	48.598	0.058	1.136
	NC				0.499	9.859^***^
Challenge	DD	0.003	0.000	0.002	-0.003	-0.043

Note^*^p < 0.05, ^**^p < 0.01; DD = daytime disturbances; NC = need for cognition

**Table 7:** Mediating effect of need for cognition between daytime disturbances on creativity

		Effect	BootSE	BootLLCI	BootULCI
Creativity	Direct	0.152	0.052	0.049	0.254
	Indirect	-0.054	0.029	-0.113	-0.001
Adventure	Direct	0.013	0.054	-0.093	0.119
	Indirect	-0.045	0.024	-0.095	-0.002
Curiosity	Direct	0.064	0.053	-0.041	0.170
	Indirect	-0.048	0.026	-0.105	0.000
Imagination	Direct	0.258	0.056	0.148	0.369
	Indirect	-0.028	0.015	-0.065	-0.003
Challenge	Direct	0.058	0.051	-0.042	0.157
	Indirect	-0.060	0.031	-0.124	-0.001

*Note* SE = standard error; LLCI = lower limit of confidence interval; ULCI = upper limit of confidence interval

## Discussion

### Insomnia and creativity in Chinese adolescents

The current study firstly examined the direct effect of insomnia (daytime disturbances, nighttime disturbances) on adolescents’ creativity. Based on our preliminary findings, insomnia was found to have a beneficial impact on the overall creativity score and imagination, aligning with prior research (H1 was supported). Further analysis showed that there indeed existed time-of-day effects: disturbances during the day had a significant effect on imagination, whereas the effect of disturbances during the night was not significant.

It’s worth nothing that the direct impact of insomnia on creativity was limited to imagination. Imagination is the ability to imagine things that have not yet happened and speculate intuitively, transcending the boundaries of the senses and reality [43]. It is the basis of all creative activities and a crucial part of culture life [44]. In a state of insomnia, individuals’ minds may be active, which may enhance individuals’ ability to visualize and increase their openness to new ideas and perspectives.

Moreover, our findings revealed a significant direct impact of daytime disturbances solely on imagination, with no comparable effect observed for nighttime disturbances. This seemed to contrast previous research conducted on young adults, indicating that ‘evening types’ - individuals who typically prefer staying up late and waking up late - tend to perform slightly better on certain measures of creativity [45]. However, it’s crucial to note that the sleep patterns of adolescents differ from those of young adults. Even if they stay up late, adolescents have less chance of waking up late. Consequently, nighttime disturbances may not be advantageous for them. Conversely, daytime disturbances resulting in fatigue and mood swings might lead to less stringent cognitive control, fostering opportunities for unconventional thinking. Hence, it becomes evident that the investigation of insomnia’s influence on creativity should take into account the time-of-day effects. Daytime disturbances appeared to positively predict creativity more strongly than nighttime disturbances in adolescents.

### Mediation of need for cognition

Although the direct impact of insomnia on creativity was notable, the majority of the observed effects were of small-to-medium magnitude. Researchers postulated the existence of a third variable that could potentially mediate the relationship between sleep and creativity [46]. To delve deeper into the influence of insomnia on creativity, we investigated the intermediary role of the need for cognition. Our findings generally indicated that insomnia might exert its influence on creativity by modulating the need for cognition (H2 was supported). The introduction of need for cognition as a variable strengthened the predictive power of insomnia-related factors (such as daytime disturbances) on creative outcomes (like imagination). These observations suggests the emergence of a suppression effect, which refers to a scenario where a third variable attenuates the relationship between an independent variable (X) and a dependent variable (Y), even when the null hypothesis is true. In psychological research, the absence of a direct relationship between X and Y often poses a challenge. The suppression effect offered a valuable framework for addressing such scenarios and elucidating why significant relationships might not be immediately apparent [47]. Similarly, our results revealed that need for cognition acted as a suppressor, mitigating the effects of insomnia on creativity.

Despite the absence of a significant direct effect of insomnia, the need for cognition was supported as an indirect influence on adventure, curiosity, and challenge. Adventurousness, curiosity, and challenge-seeking all involve cognitive endeavors such as facing failure or criticism, inquiring into the root cause of problems, engaging in confusing situations, and making order out of chaos [43]. These creative personalities are strongly influenced by their need for cognition, and insomnia may influence them indirectly by altering their need for cognition.

### Limitations

Although these findings offer valuable insights, it’s important to acknowledge several limitations. Firstly, the study employed a cross-sectional design, which assessed variables simultaneously, thus lacking evidence of a temporal link between insomnia and creativity. Longitudinal studies are needed to establish a definitive cause-and-effect relationship between these variables. Secondly, the current study primarily focused on creative personality, overlooking the impact of creative cognition. Given that insomnia is a small-to-medium predictor of divergent thinking [18], it’s crucial to investigate whether the need for cognition mediates this relationship, enhancing our understanding of the factors that truly influence insomnia’s predictive power over creativity. Finally, the findings of this study have not been replicated in other samples, limiting their generalizability. Future research should aim to replicate these results in diverse enrollment groups, particularly those experiencing severe insomnia, to gain a more comprehensive understanding of the phenomenon.

### Conclusions

Despite the limitations described, the present study has two strengths. The primary strength is to first reveal the time-of-day effect associated with insomnia and adolescents’ creativity. These preliminary findings offer profound insights into the impact of sleep disturbances on adolescents’ creativity, thereby aiding in the development of accurate sleep concepts and promoting mental well-being. Secondly, insomnia was found to be more likely to influence creativity through affecting need for cognition. These revelations contribute to establishing scientific frameworks for understanding adolescents’ sleep patterns and suggest that the need for cognition is a crucial aspect in examining the link between insomnia and creativity. Notably, the suppression effect of the need for cognition offers an explanation for the tenuous association between insomnia and creativity, providing a theoretical foundation for fostering the emergence and development of creativity among adolescents with insomnia.

### Electronic supplementary material

Below is the link to the electronic supplementary material.

Supplementary Material 1

## Data Availability

Data is provided within the manuscript.

## Abbreviations

WCTS: Williams Creative Tendencies Scale
NCS: Need for Cognition Scale
BIS: Bergen Insomnia Scale
SES: Socioeconomic Status
CMV: Common Method Variance
SES_f_: Father Socioeconomic Status
SES_m_: Mother Socioeconomic Status
DD: Daytime Disturbances
ND: Nighttime Disturbances
NC: Need for Cognition
SE: Standard Error
