# Supplementary material for: Insomnia and creativity in Chinese adolescents: mediation through need for cognition
Source: BMC Psychol. 2024 Mar 29;12:180. doi: 10.1186/s40359-024-01663-3 (PMC10981307; doi:10.1186/s40359-024-01663-3)
Supplement: Supplementary file 1 — Supplementary Material 1 [file 40359_2024_1663_MOESM1_ESM.docx]

**Table S1** Indirect effect of need for cognition between nighttime disturbances on creativity

|  |  | Model fit | | | Regression coefficient | |
| --- | --- | --- | --- | --- | --- | --- |
| Outcome | Predictor | R | R^2^ | F | β | t |
| NC | ND | 0.103 | 0.011 | 3.198 | -0.103 | -1.788 |
| Creativity | ND | 0.427 | 0.183 | 33.402 | 0.012 | 0.234 |
|  | NC |  |  |  | 0.428 | 8.151^***^ |
| Creativity | ND | 0.032 | 0.001 | 0.302 | -0.032 | -0.549 |
| NC | ND | 0.103 | 0.011 | 3.198 | -0.103 | -1.788 |
| Adventure | ND | 0.374 | 0.140 | 24.363 | -0.036 | -0.663 |
|  | NC |  |  |  | 0.369 | 6.844^***^ |
| Adventure | ND | 0.074 | 0.005 | 1.635 | -0.074 | -1.279 |
| NC | ND | 0.103 | 0.011 | 3.198 | -0.103 | -1.788 |
| Curiosity | ND | 0.394 | 0.155 | 27.384 | -0.023 | -0.430 |
|  | NC |  |  |  | 0.390 | 7.305^***^ |
| Curiosity | ND | 0.063 | 0.004 | 1.199 | -0.063 | -1.095 |
| NC | ND | 0.116 | 0.014 | 1.356 | -0.100 | -1.712 |
| Imagination | ND | 0.330 | 0.109 | 9.091 | 0.104 | 1.883 |
|  | NC |  |  |  | 0.193 | 3.491^***^ |
| Imagination | ND | 0.269 | 0.073 | 7.768 | 0.085 | 1.518 |
| NC | ND | 0.103 | 0.011 | 3.198 | -0.103 | -1.788 |
| Challenge | ND | 0.494 | 0.244 | 48.209 | -0.042 | -0.836 |
|  | NC |  |  |  | 0.488 | 9.646^***^ |
| Challenge | ND | 0.092 | 0.009 | 2.582 | -0.092 | -1.607 |

Note: ^*^*p* < 0.05, ^**^*p* < 0.01; NC= need for cognition; ND=nighttime disturbances.

**Table S2** Mediating effect of need for cognition between nighttime disturbances on creativity

|  |  | Effect | BootSE | BootLLCI | BootULCI |
| --- | --- | --- | --- | --- | --- |
| Creativity | Direct | 0.012 | 0.053 | -0.091 | 0.116 |
|  | Indirect | -0.044 | 0.027 | -0.098 | 0.007 |
| Adventure | Direct | -0.036 | 0.054 | -0.142 | 0.070 |
|  | Indirect | -0.038 | 0.024 | -0.088 | 0.006 |
| Curiosity | Direct | -0.023 | 0.053 | -0.128 | 0.082 |
|  | Indirect | -0.040 | 0.025 | -0.093 | 0.005 |
| Imagination | Direct | 0.104 | 0.055 | -0.005 | 0.213 |
|  | Indirect | -0.019 | 0.013 | -0.052 | 0.002 |
| Challenge | Direct | -0.042 | 0.051 | -0.142 | 0.057 |
|  | Indirect | -0.050 | 0.030 | -0.111 | 0.007 |

Note: SE=standard error; LLCI=lower limit of confidence interval; ULCI=upper limit of confidence interval.
